# FACT in Cell Differentiation and Carcinogenesis

**DOI:** 10.18632/oncotarget.356

**Published:** 2011-11-16

**Authors:** Fu-Kai Hsieh, Olga I. Kulaeva, Igor V. Orlovsky, Vasily M. Studitsky

**Affiliations:** ^1^ Department of Pharmacology, UMDNJ-Robert Wood Johnson Medical School, Piscataway, NJ, USA; ^2^ Faculty of Biology, Moscow State University, Moscow, Russia

Recent work of several laboratories has identified FACT (facilitates chromatin transcription) protein complex as an emerging target for development of anticancer drugs and suggested a mechanism of FACT action during transcription and cell differentiation.

FACT is the transcription and replication factor [1], which can stimulate transcript elongation through chromatin *in vitro* [2, 3]. Human FACT is a heterodimer composed of two subunits Spt16 and SSRP1 and has a histone chaperone activity [3, 4]. *In vivo*, FACT and RNA polymerase II (Pol II) co-localize and display similar kinetics of recruitment and chromosome tracking [5, 6]. FACT also maintains the nucleosome integrity during transcription initiation and transcript elongation by Pol II [6-9]. Currently, two different models have been proposed to explain the functions of FACT to nucleosomes. On one hand, it has been suggested that FACT-assisted histone H2A/H2B dimer dissociation from nucleosome facilitates transcription through chromatin [3] and perhaps chromatin re-assembly. On the other hand, the studies of yeast FACT suggested that it can globally affect the nucleosome structure to allow access of regulatory proteins to histone-covered DNA [10, 11].

In a recent careful biochemical study from laboratory of K. Luger [12] interactions between FACT and its various potential interaction targets in chromatin (histones H2A-H2B and H3-H4, histone tails, nucleosomal and linker DNA) were quantified. It was shown that FACT preferentially binds to H2A-H2B dimer and this high-affinity binding is largely mediated by the acidic C-terminal domain of the Spt16 subunit. The observation that FACT competes with DNA for a shared interaction interface on H2A-H2B dimer suggests a possible mechanism of FACT-facilitated DNA displacement from the histone octamer and possibly subsequent nucleosome recovery. Indeed, as various processive DNA-targeted enzymes (e.g. polymerases and ATP-dependent chromatin remodelers) encounter nucleosomes and attempt to displace histones from DNA, progression of the enzymes should be greatly facilitated by the FACT-dimer interactions. The recovery of chromatin structure could be facilitated by chaperone activity of FACT though facilitated re-binding of the dimers to chromatin or their more efficient survival during transcription, as was suggested recently [13].

Analysis of FACT expression in various tissues using immunohistochemical methods and NCBI Gene Expression Omnibus data analysis (laboratory of K. Gurova) have shown that the SSRP1 and SPT16 subunits are expressed in a coordinated manner, and the levels of expression are highly variable within the tissues [14]. The levels of expression are higher in non-differentiated and cancer cells, and do not correlate with expression of the Ki67 proliferation marker, suggesting that that FACT expression is more related to differentiation than to proliferation. Induction of oncogenes in different cells in most cases results in an increased expression of FACT subunits. Furthermore, tumor cells are sensitive to FACT downregulation, suggesting that FACT could be an important new target for development of anti-cancer drugs.

The latter line of research has been extended in another work from Gurova group [15]. It was shown that cancer cells, like undifferentiated cells, contain higher levels of FACT. Small DNA-intercalating molecules, curaxins, have a strong anticancer activity, activate p53 and inhibit NF-kB without causing detectable genotoxicity. The effects of curaxins on p53 and NF-kB, as well as their toxicity to cancer cells, result from “chromatin trapping” of FACT (depletion of soluble, functionally active FACT), presumably through interaction with DNA-bound curaxins that is particularly detrimental for cancer cells. Trapping of FACT leads to phosphorylation of the p53 by casein kinase 2 (CK2) and inhibition of NF-kB-dependent genes that require FACT activity during transcript elongation stage.

**Figure 1:**
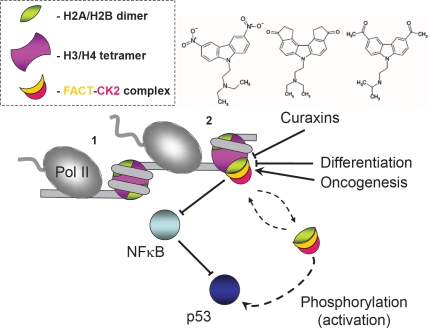
FACT as a regulatory hub in cell differentiation and carcinogenesis As RNA polymerase II (Pol II) encounters nucleosomes (1) and uncoils nucleosomal DNA from core histones (2), FACT interacts with the H2A/H2B dimer and facilitates transcription through chromatin. Binding of FACT results in cell growth inhibition through inhibition of NK-κB and activation of p53 by CK2 phosphorylation. Curaxins intercalate into the DNA, induce a conformational change in the DNA and interfere with recruitment of FACT.

How can the changes in FACT content during cell differentiation and carcinogenesis be rationalized in the framework of the current models of FACT action? Since FACT works primarily during transcript elongation step (likely via interactions with H2A/H2B dimers, Fig. 1), it is required in large amounts in cells where gene expression occurs at a higher level, such as cancer or undifferentiated cells. Therefore these cells are more sensitive to inhibitors affecting expression or activity of FACT. Indeed, recent proteomic studies of Xiong *et al*. [16] suggest that proteins involved in DNA replication, chromatin remodeling, and Pol II-dependent transcription are down-regulated in differentiated cells. Future analysis of the molecular mechanisms of FACT action and its interaction with anticancer drugs should reveal whether FACT expression plays a regulatory role in differentiation and carcinogenesis, and should result in development of more efficient cancer therapies.
